# A UNIQUE ADIPOCYTE PROGENITOR POPULATION PROMOTES AGE-RELATED ADIPOSITY

**DOI:** 10.1093/geroni/igac059.1263

**Published:** 2022-12-20

**Authors:** Qiong (Annabel) Wang

**Affiliations:** City of Hope, City of Hope, California, United States

## Abstract

The average fat mass in adults increases dramatically with age, and older people often suffer from visceral obesity and related adverse metabolic disorders. Unfortunately, how aging leads to fat accumulation is poorly understood. It is known that fat cell (adipocyte) turnover is very low in young mice, similar to that in young humans. Here, we find that mice mimic age-related fat expansion in humans. In vivo lineage tracing shows that massive adipogenesis (the generation of new adipocytes), especially in the visceral fat, is triggered during aging. Thus, in contrast to most types of adult stem cells that exhibit a reduced ability to proliferate and differentiate, the adipogenic potential of adipocyte progenitor cells (APCs) is unlocked by aging. In vivo transplantation and 3D imaging of transplants show that APCs in aged mice cell-autonomously gain high adipogenic capacity. Single-cell RNA sequencing analyses reveal that aging globally remodels APCs. Herein, we identify a novel committed preadipocyte population that is age-specific (CP-A), existing both in mice and humans, with a global activation of proliferation and adipogenesis pathways. CP-A cells display high proliferation and adipogenesis activity, both in vivo and in vitro. LIFR serves as a functional maker of CP-A, which promotes CP-A proliferation. Together, these findings define a new fundamental mechanism involved in fat tissue aging and offer prospects for preventing and treating age-related metabolic disorders.

